# Complex spatiotemporal haemodynamic response following sensory stimulation in the awake rat^☆^

**DOI:** 10.1016/j.neuroimage.2012.10.006

**Published:** 2013-02-01

**Authors:** Chris Martin, Ying Zheng, Nicola R. Sibson, John E.W. Mayhew, Jason Berwick

**Affiliations:** a Department of Psychology, University of Sheffield, Western Bank, Sheffield, S10 2TP, UK; b Radiobiology Research Institute, Department of Oncology, University of Oxford, Churchill Hospital, Headington, Oxford, OX3 7LJ, UK

**Keywords:** fMRI, Neurovascular coupling, Anaesthesia, Haemodynamic, Optical imaging

## Abstract

Detailed understanding of the haemodynamic changes that underlie non-invasive neuroimaging techniques such as blood oxygen level dependent functional magnetic resonance imaging is essential if we are to continue to extend the use of these methods for understanding brain function and dysfunction. The use of animal and in particular rodent research models has been central to these endeavours as they allow in-vivo experimental techniques that provide measurements of the haemodynamic response function at high temporal and spatial resolution. A limitation of most of this research is the use of anaesthetic agents which may disrupt or mask important features of neurovascular coupling or the haemodynamic response function. In this study we therefore measured spatiotemporal cortical haemodynamic responses to somatosensory stimulation in awake rats using optical imaging spectroscopy. Trained, restrained animals received non-noxious stimulation of the whisker pad via chronically implanted stimulating microwires whilst optical recordings were made from the contralateral somatosensory cortex through a thin cranial window. The responses we measure from un-anaesthetised animals are substantially different from those reported in previous studies which have used anaesthetised animals. These differences include biphasic response regions (initial increases in blood volume and oxygenation followed by subsequent decreases) as well as oscillations in the response time series of awake animals. These haemodynamic response features do not reflect concomitant changes in the underlying neuronal activity and therefore reflect neurovascular or cerebrovascular processes. These hitherto unreported hyperemic response dynamics may have important implications for the use of anaesthetised animal models for research into the haemodynamic response function.

## Highlights

► Rats were trained to undergo optical imaging spectroscopy whilst fully conscious. ► Haemodynamic responses to whisker pad stimulation were recorded. ► Responses contained oscillations and inversions not found in anaesthetised animals. ► The results may be important for in-vivo research into brain imaging signals.

## Introduction

The haemodynamic response to alterations in neuronal activity underlies the blood oxygen level dependent (BOLD) signals which are used in fMRI to non-invasively study task or drug dependent changes in brain function. Although fMRI has become an immensely important tool in basic cognitive neuroscience and may become increasingly valuable in clinical research, as a technique it suffers from intrinsically low signal to noise relative to direct measurements of neuronal activity (Logothetis et al., 2001) and as such sophisticated analysis tools are required to extract spatiotemporal signal changes attributable to the experimental manipulations. These analysis tools typically incorporate canonical models of the haemodynamic response function (HRF), and as such a detailed understanding of the spatiotemporal characteristics of the HRF is critically important for the analysis and interpretation of functional imaging data (e.g. Buxton and Frank, 1997; Worsley et al., 2002; Zheng et al., 2005). Much of the work conducted to provide this detailed understanding to date has used animal models and optically based techniques since these typically afford far greater temporal and spatial resolution than is currently possible using fMRI (e.g. Boorman et al., 2010; Tian et al., 2010). Since these techniques are invasive and to minimise movement artefacts in the imaging data, almost all of this research has been conducted in anaesthetised animals. Notwithstanding species-related considerations in the translation of data acquired in these studies to human functional brain imaging, anaesthesia itself has previously been shown to alter the temporal dynamics of the haemodynamic impulse response function (Martin et al., 2006a) and is known to impact upon neurovascular coupling and vascular reactivity (see discussion in Martin et al., 2006a; also Masamoto et al., 2009; Liu et al., 2012). To address this limitation, we established optical imaging methods to investigate in detail the spatiotemporal haemodynamic response function in un-anaesthetised rats (Martin et al., 2002, 2006a, 2006b). Here, we show that the cortical haemodynamic response to a somatosensory stimulus in awake rats contains spatiotemporal features hitherto unreported in studies with comparable resolution, in which anaesthetised animals have been used.

## Material and methods

All animal procedures were carried out with the approval of both the University of Sheffield local ethics committee and the UK Home Office under the Animals (Scientific Procedures) Act 1986. Animals were handled extensively and trained to accept comfortable head and body restraint prior to surgical preparation and imaging procedures (see below and Martin et al., 2002). In all cases, animals were female hooded Lister rats weighing between 200 and 300 g, kept in a 12-h dark/light cycle environment at a temperature of 22 °C with food and water ad libitum. The haemodynamic data were collected from 18 experimental sessions using 7 animals (2–4 sessions per animal), whilst the neuronal response data were collected from 16 experimental sessions using 5 animals.

Animals were anaesthetised with an intraperitoneal injection of ketamine and xylazine (1.1 ml/kg). The skull was exposed and a section overlying somatosensory cortex thinned to translucency. For animals used for electrophysiological recording, a 16-channel micro-electrode array was then inserted into the barrel cortex under stereotaxic control, and secured in place using cyanoacrylate and dental cement. For animals to be used for optical imaging, a coating of clear cyanoacrylate was applied to the thin cranial window to increase strength. Bone screws were then secured into burr holes anterior and posterior to the thinned skull. An imaging chamber was then secured in layers of dental cement. Teflon coated tungsten microwires (diameter 0.2 mm, Advent Research Materials Ltd., Oxford, UK) were chronically implanted into the contralateral whisker pad during surgery for subsequent stimulus delivery. The wires were fed subcutaneously to a connector set in dental cement adjacent to the imaging chamber, which was coupled to the stimulator device during experimental sessions. All wounds were closed and the animals were treated with an analgesic (Rimadyl, 0.05 ml, s.c.) for 24 h after surgery. Animals were then left to recover for 3–5 days.

**Fig. 1:**
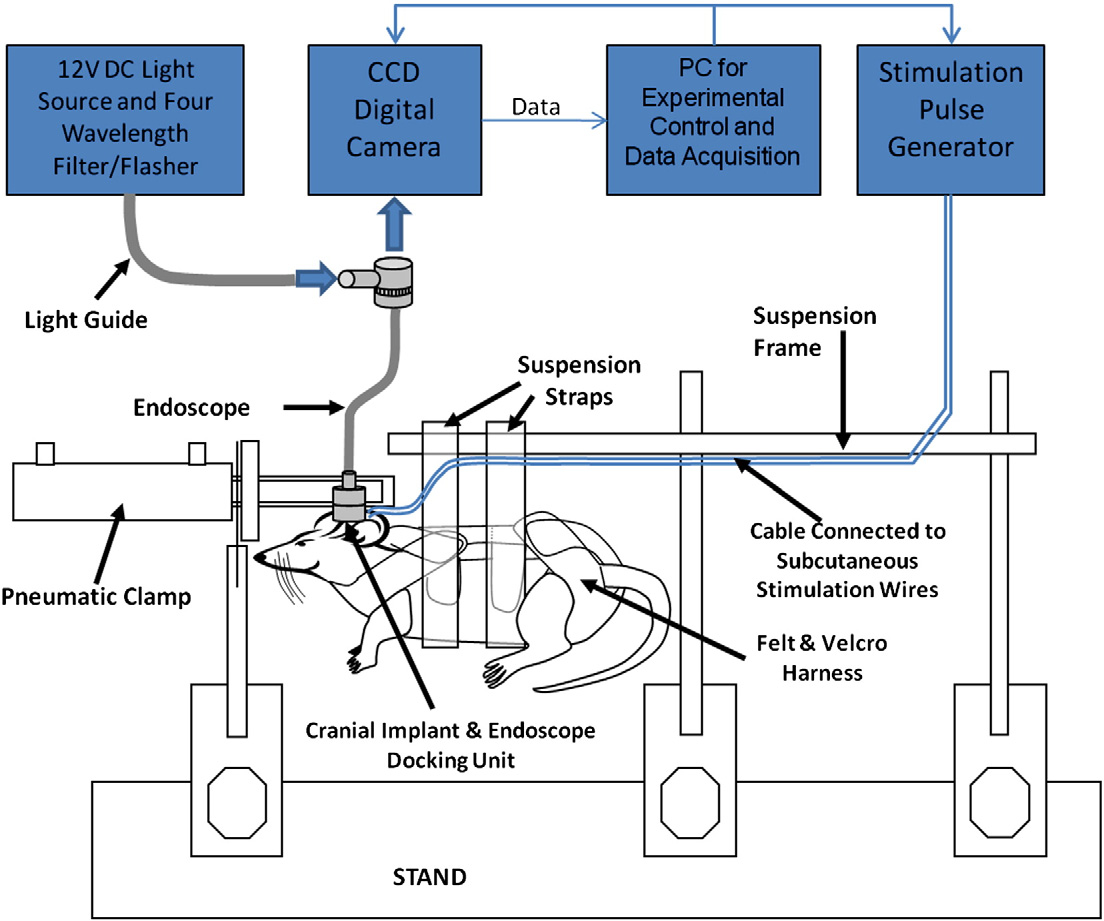
Illustration of experimental apparatus for optical imaging in un-anaesthetised rats. Trained animals were first restrained in the harness and the head was stabilised by clamping the chronically implanted imaging chamber. The endoscope was then attached to the implanted chamber and connections were made to the subcutaneously implanted stimulation wires.

For each experiment, animals were placed into a harness which was suspended from a frame. To reduce head movements, a pneumatically operated clamp secured the implanted imaging chamber and therefore the head. The experimental apparatus is illustrated in Fig. 1. The imaging chamber was filled with warm saline and a 3 mm endoscope was positioned in the chamber to provide both illumination of the cortex and transmission of the remitted images to a digital camera. Procedures were identical for animals undergoing electrophysiological recording, except that once secured in the restraint apparatus the implanted electrode was connected to a preamplifier unit which relayed signals to the data acquisition device (Medusa Bioamp', Tucker Davis Technology, Florida).

Stimulation of the whisker pad consisted of a 16-s, 5 Hz pulse train with an individual pulse width of 0.3 ms. This stimulation duration was chosen to enable us to investigate response dynamics that occur beyond the initial haemodynamic ‘impulse response’, and to be more comparable with typical human neuroimaging stimulation parameters. Longer stimuli were not used due to the increases in time-to-recover and the consequences for total duration of imaging sessions in awake animals. A stimulation frequency of 5 Hz was chosen as this represents a frequency which has been shown to produce robust responses in awake animals and across a wide range of anaesthetic regimes. Data were collected for 8-s preceding stimulus onset to provide baseline measures and for 40-s following stimulus onset to capture activation induced haemodynamic changes and the return to baseline following stimulus offset. Each experimental session consisted of 30 trials. Stimulus amplitude was set at approximately 0.4 mA which has previously been shown to produce robust haemodynamic responses in awake animals with no indication of being noxious or producing stimulus-locked movements of the animal (Martin et al., 2006a). Further details of the awake animal training, preparation and data collection procedures can be found in Martin et al. (2002, 2006a, 2006b).

At the end of the last awake imaging session, 6 animals were anaesthetised with an intraperitoneal injection of urethane (1.25 g/kg, ip), tracheotomised and cannulated for monitoring and maintenance of physiological parameters (as described previously in Martin et al., 2006a; Berwick et al., 2008). The stimulus amplitude was increased to 1.2 mA in order to produce robust responses in the anaesthetised state (Martin et al., 2006a).

To generate spatiotemporal data for spectral analysis, the cortex was illuminated at 4 wavelengths of light (495, 586, 559 and 575 nm) using a Lambda DG-4 high-speed filter changer light source (Sutter Instrument Company, Novato, CA, USA). Remitted images passed into a 12-bit CCD camera (SMD 1 M30). The frame rate of the camera was 32 Hz which was synchronised to the filter switching, thereby giving an 8-Hz effective frame rate. The spectral analysis was based upon the path length scaling algorithm (PLSA) described previously (Berwick et al., 2005). Baseline blood haemoglobin concentration (HbT_0_) and oxygenation (O_2_Sat_0_) were estimated on a pixel by pixel basis using pre-stimulus absorption data. To constrain estimated values within physiologically plausible limits, this step in the analysis assumed average values across the visible cortex to be 104 μMol (HbT_0_) and 50% (O_2_Sat_0_), based on Kennerley et al. (2005). The spectral analysis yielded spatiotemporal maps of changes in oxyhaemoglobin (HbO_2_), deoxyhaemoglobin (Hbr) and total haemoglobin (Hbt) concentration. Data were trial-averaged to yield a single dataset for each experimental session.

To determine the neuronal responses to the stimulation paradigm used in this study, local field potential (LFP) and multi-unit activity (MUA) recordings were made from barrel cortex of a separate group of awake animals under comparable experimental conditions using chronically implanted multichannel electrodes. Response data were taken from a recording site corresponding to layer IV of the barrel cortex (depth of approximately ~ 450 μm). To produce an estimate of MUA, data were high pass filtered (> 300 Hz), thresholded (> 3SD) and ‘spikes’ counted in bins corresponding to the 80 stimulus pulses delivered by this stimulation train.

## Results

**Fig. 2:**
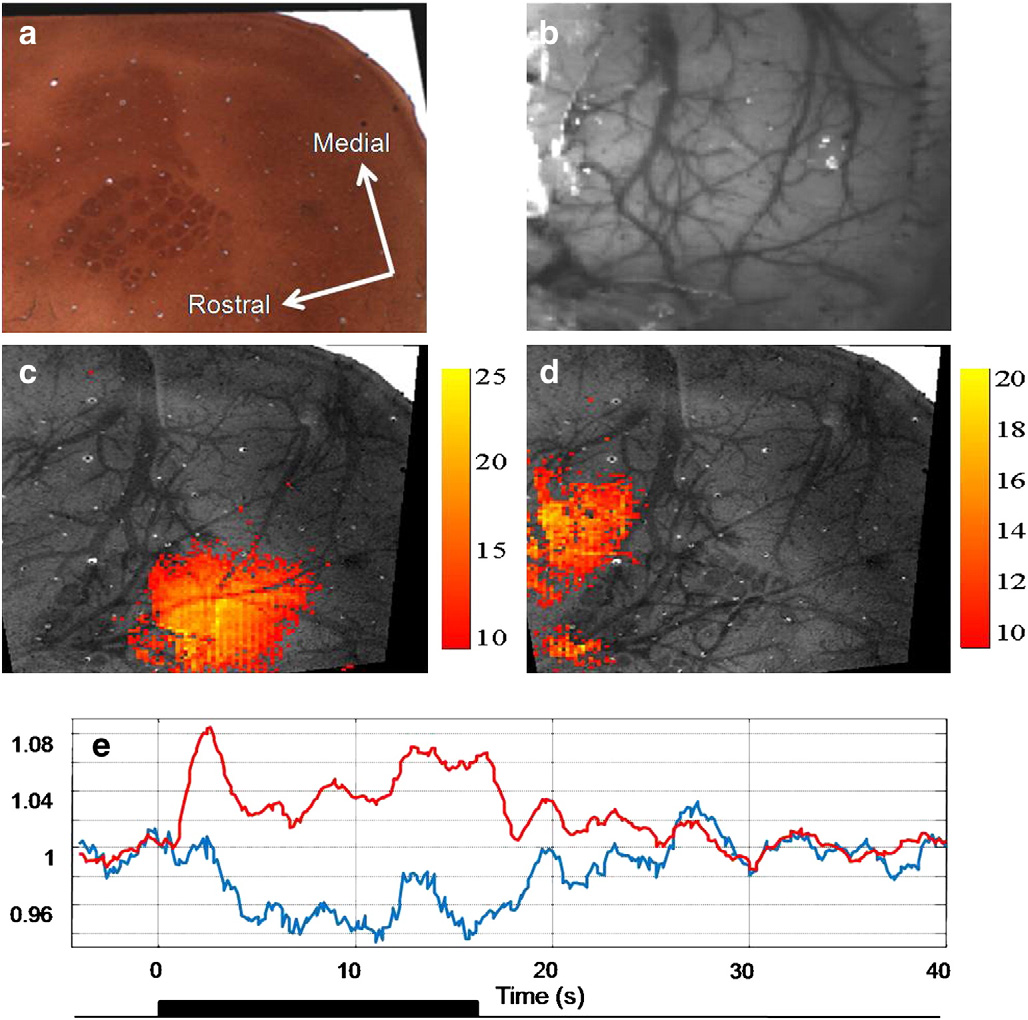
Correspondence of anatomy and functional activation. (a) Cytochrome oxidase stained section of somatosensory cortex showing whisker barrels. (b) Image of cortex and surface vasculature acquired during optical imaging experiments. Image obtained through thinned skull of an awake animal 6 days after surgical preparation (c & d) a composite image is formed by registering the histological image to the experimental image using penetrating blood vessels (see Material and methods). Activation data (based on changes in oxyhaemoglobin concentration) can then be overlayed. Activation images were obtained by correlating time series changes for each pixel with the stimulus (shown in (e)), where (c) depicts positive correlation (normal response region) and (d) depicts negative correlation (inverted response region). Scale bar represents z-score of ‘activated’ pixels. (e) Time series of oxyhaemoglobin concentration changes in each of the response regions (c — red line; d — blue line).

**Fig. 3:**
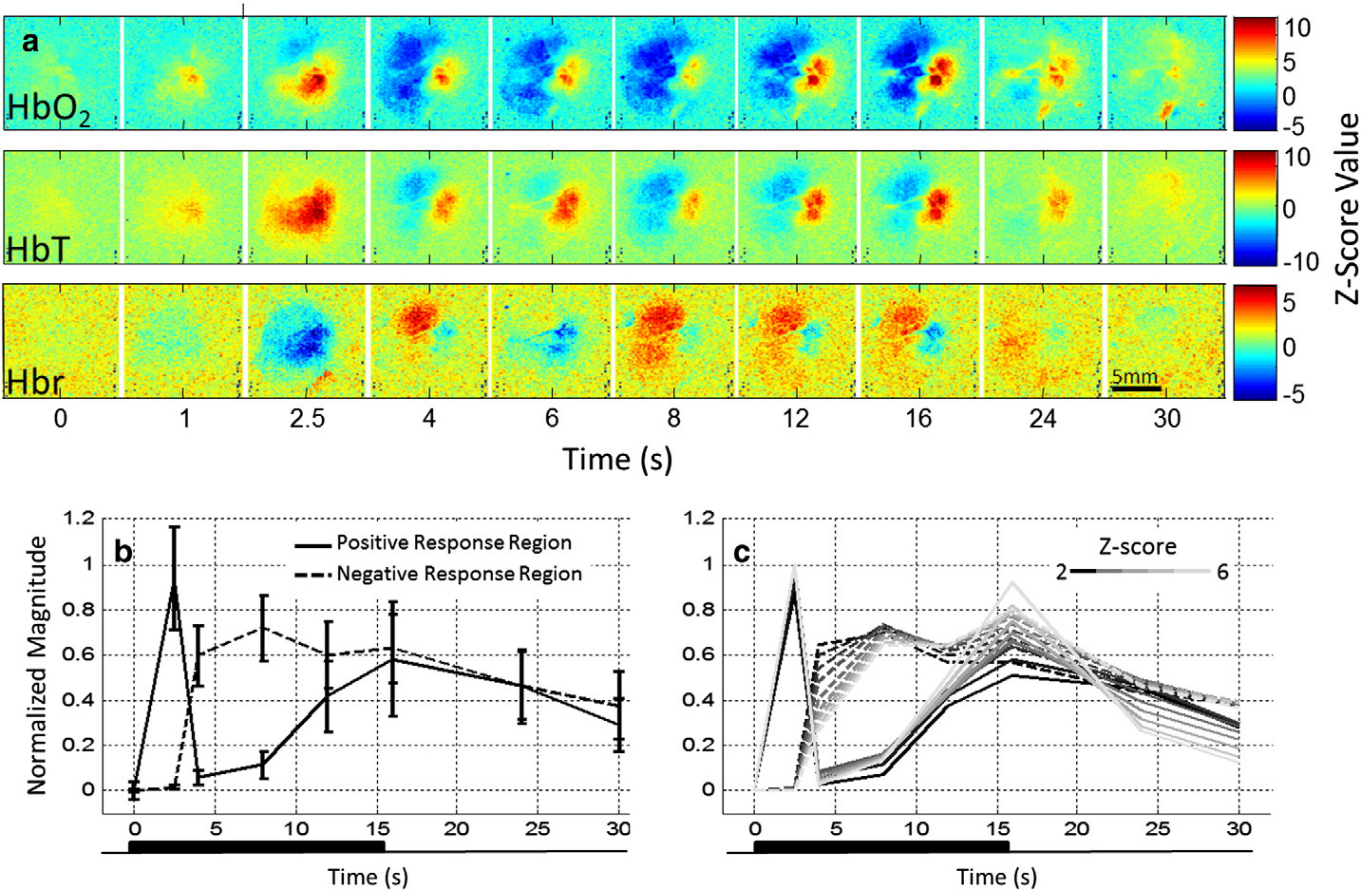
(a) Spatiotemporal changes in the concentration of oxy-, deoxy- and total-haemoglobin in rat somatosensory cortex during a 16 s electrical stimulation of the contralateral whisker pad (5 Hz, 0.4 mA). Data were converted to z-scores on a pixel by pixel basis using the mean and standard deviation of the 8 s pre-stimulus baseline. (b) Spatial extent of haemodynamic response. Changes in size (number of pixels) of cortical area where oxyhaemoglobin concentration increases (normal response, solid lines) or decreases (inverted response, dashed lines) exceed a z-score threshold of +/− 3. Trial-averaged data from each animal were normalised between 0 (corresponding to baseline at t = 0) and 1 (corresponding to the maximum number of pixels displaying oxyhaemoglobin increases). Data were then averaged across animals. Error bars denote standard error of the mean. (c) To illustrate the effect of z-score threshold on the spatial extent of the response, data were plotted as in (b) but for a range of z-scores (error bars omitted for clarity).

Electrical stimulation of the whisker pad produces changes in haemoglobin concentration and oxygenation in the imaged contralateral somatosensory cortex of awake rats (Figs. 2 and 3a, representative data). The expected haemodynamic response (hereafter: ‘positive response’) to stimulus-induced increases in neural activity comprises of an increase in HbO_2_ and HbT, and a decrease in Hbr concentration, as freshly oxygenated blood perfuses the imaged tissue, ‘washing out’ deoxyhaemoglobin. Soon after stimulation onset, this response profile was evident over a large area of imaged cortex (Fig. 3, t = 2.5 s). Subsequently, the spatial extent of this response became restricted to a much smaller area (Fig. 3, t = 4–16 s). In surrounding cortex, an inverted response (hereafter: ‘negative response’), consisting of decreases in HbO_2_ and HbT, and increases in Hbr concentration was observed. This negative haemodynamic response did not become evident until 4.0 s into the stimulation period, but was then sustained over a larger area of the imaged cortex until stimulation ceased (Fig. 3). After stimulation offset, all haemodynamic changes decreased in magnitude and spatial extent as they returned to baseline, with the negative responses resolving more quickly. To compare changes in the spatial extent of the above and below baseline haemodynamic changes and determine the consistency of these features across animals, counts of pixels exceeding an activation threshold (where − 3 > z > 3) were made for a number of time points on each dataset after trial-averaging (Fig. 3b). This analysis confirms that the spatiotemporal changes described above and illustrated in Fig. 3a were consistent across the data sets. Specifically, the positive response to stimulation was initially evident over a larger area than the negative response during the first few seconds of the stimulation. Subsequently however, the spatial extent of the negative response increased to approximately 5 times that of the positive response. Another consistent feature was that whilst the negative response area reached a plateau during stimulation, the positive response area continued to expand. To show the effect of choice of z-score threshold on the temporal changes in response areas, the pixel count was repeated for a range of z-score threshold values (Fig. 3c). The main differences in the temporal changes in the activation area between the positive and negative haemodynamic responses appear to be independent of the choice of activation z-score threshold.

Time series data were extracted from the spatiotemporal data by selecting regions of interest (ROIs) for each data set from the haemodynamic response image 16 s after stimulation onset (Fig. 3a), at which time point the spatial responses had stabilised (note the response oscillations discussed below). Regions were selected to include the maximally responsive regions for each of the two types of response identified above: (1) positive responses, where the principal haemodynamic changes consisted of increased HbO_2_ and HbT concentration and decreased Hbr concentration; (2) negative responses, where the principal haemodynamic changes consisted of decreased HbO_2_ and HbT concentration and increased Hbr concentration. Consistently sized ROIs were selected (ROI size, in mm^2^: positive response, 1.11 +/− 0.12; negative response, 1.04 +/− 0.11), with no significant difference between the sizes of the ROIs chosen for the positive or negative response (t = 1.277, df = 17, p < 0.219).

**Fig. 4:**
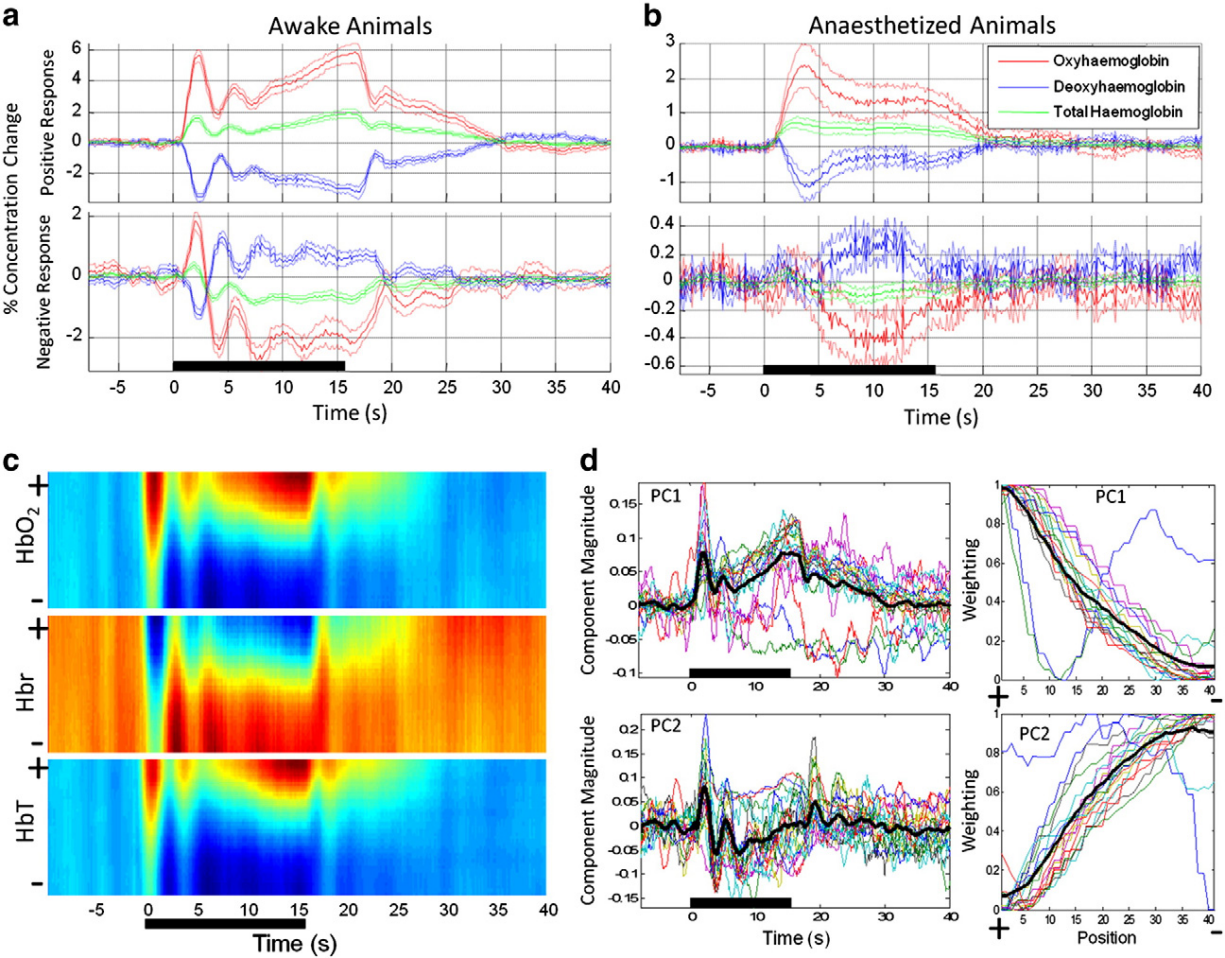
Cortical haemodynamic responses to a 16 s stimulation of the contralateral whisker pad. (a) Time series of trial-averaged signal changes recorded in awake animals from positive (top) and negative (bottom) response regions. (b) As (a), but for anaesthetised animals. Time series represent percentage changes from pre-stimulus baseline and dotted lines show +/− standard error of the mean. (c) Spatiotemporal plots of the mean time series of changes in HbO_2_, Hbr and HbT from linearly spaced regions of interest lying between the previously identified positive (top, +) to negative (bottom, −) response regions. The figure shown is the averaged spatiotemporal response map across all datasets. (d) First two components from a principal components (PC) analysis of each dataset (top, PC1; bottom, PC2). The time series (eigenvectors) for the first two components calculated for all 18 datasets is shown (left) along with the weighting of these components (eigenvalues) in terms of position between the positive and negative response regions (right). The mean eigenvectors and eigenvalues are overlayed in black.

Time series data from each ROI representing fractional changes from pre-stimulus baseline values were extracted from each data set and then averaged across the 18 experimental sessions (Fig. 4a). Initially HbO_2_ and HbT concentration increases rapidly to maxima of 6.01 ± 0.36% and 1.75 ± 0.16% with latencies of 2.52 ± 0.07 s and 2.25 ± 0.09 s respectively while Hbr concentration decreases by 4.02 ± 0.2% at 2.69 ± 0.08 s. After this initial response component, there is a partial return towards baseline before secondary increases in HbO_2_ and HbT and decreases in Hbr. The second positive response peak is approximately 2/3 the size of the first and occurs 3–3.5 s later. Close inspection suggests subsequent, smaller oscillations in the time series, although after the second positive response peak the dominant feature is a monotonic increase in response magnitude until stimulation offset (at 16 s), whereupon the time series returns to baseline (Fig. 4a). The initial component of the negative response time series is similar to that of the positive response, with rapid increases in HbO_2_ and HbT concentration to maxima of 2.16 ± 0.29% and 0.52 ± 0.08% with latencies of 2.16 ± 0.11 s and 1.75 ± 0.15 s respectively while Hbr concentration decreases by 1.60 ± 0.15% at 2.37 ± 0.13 s. This response component is approximately 1/3 the magnitude of the initial positive response peak and occurs earlier (0.36 s, 0.50s and 0.32 s for HbO_2_, Hbr and HbT respectively; HbO_2_: t = 2.83, df = 17, p < 0.05; HbT: t = 2.67, df = 17, p < 0.05). There is then an inversion of the negative response time series where the HbO_2_ and HbT components decrease below baseline, whilst the Hbr component increases above baseline. The negative response time series then continue to oscillate until stimulation ends, whereupon they return rapidly to baseline (Fig. 4a).

The experiments were repeated in a subgroup of animals placed under anaesthesia after the final un-anaesthetised data collection session and the time series of the responses extracted from positive and negative response regions of interest in these data is plotted in Fig. 4b. The main features of these responses contrast markedly with those observed in the awake animals. Specifically the oscillatory components, response inversion and monotonically increasing response magnitude were not present. The initial HbO_2_ and HbT concentration increase peaks at 2.81 ± 0.64% and 0.82 ± 0.02% with latencies of 3.78 ± 0.23 s and 3.33 ± 0.30s respectively whilst Hbr concentration decreases by 1.65 ± 0.36% at 4.38 ± 0.25 s. Quantitation of time series features recorded from the negative response regions is not presented for anaesthetised animals due to the very low amplitude of these responses (the time series is shown for completeness).

To further investigate the spatiotemporal response profile we extracted additional time series from regions of interest linearly spaced between the ROIs chosen for the positive and negative responses. These spatiotemporal ‘transition maps’ were normalised between 0 and 1 for each experimental session and the average of these maps is plotted in Fig. 4c. A principal components analysis on each data set (Fig. 4d) revealed the first component explained on average 81.9% (± 10.0) and of the variance in the data, and the addition of the second component increased the amount of variance explained to an average of 99.4% (± 0.68). The first and second principal components and their eigenvectors are plotted in Fig. 4d. These two components appear very similar to the time series for the positive and negative response regions of interest.

**Fig. 5:**
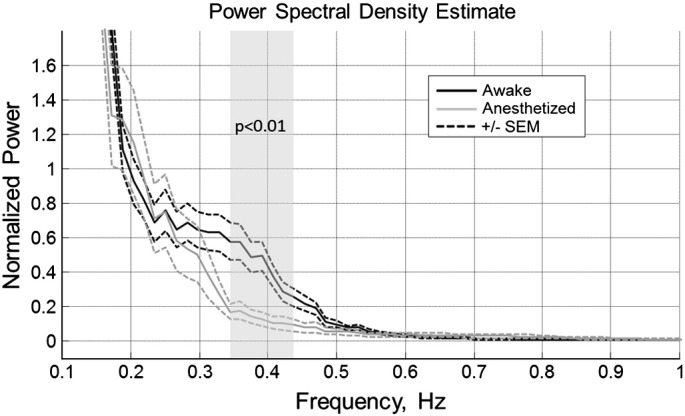
Normalised power spectral density estimate of the time series recorded from the positive response regions of awake and anaesthetised animals. Error bars (dashed lines) denote standard error of the mean. The shaded area indicates the frequency values where a significant difference (p < 0.01) in power amplitude is found between the anaesthetic conditions, computed by unpaired t-tests on each data point.

To further explore the differences in the response dynamics apparent from the time series presented in Figs. 3–4, we computed power spectral density estimates for the HbT responses in both the awake and anaesthetised animal datasets. The normalised power spectra are reported in Fig. 5, which shows a difference in power over the frequency range ~ 0.35 Hz–0.45 Hz. This difference corresponds well to the timing of the more rapid and oscillatory response characteristics observed in the awake animal data, but not in the anaesthetised animal data.

**Fig. 6:**
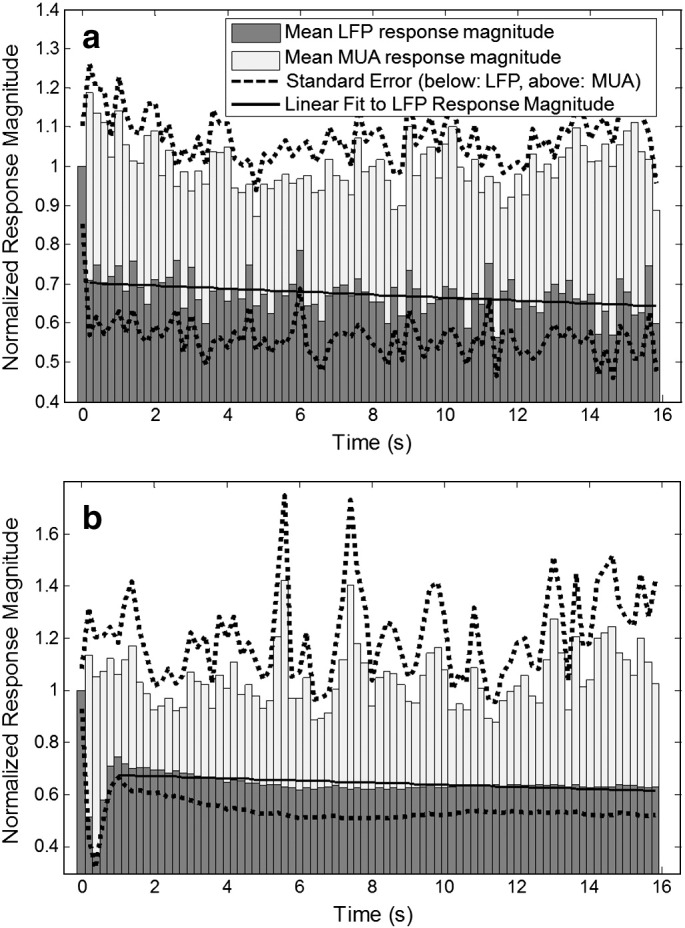
Average, normalised, LFP (dark grey) and MUA (light grey) response magnitudes recorded from barrel cortex of (a) awake and (b) anaesthetised rats in response to a 16 s, 5 Hz stimulation of the whisker pad via chronically implanted stimulating electrodes. The solid line represents a 1st order fit to the LFP data, excluding the first pulse (slope = − 0.039; r = 0.382; p < 0.001). Dashed lines denote standard error of the mean, shown only above the MUA data and below the LFP data for clarity. The data for anaesthetised animals was previously reported in Boorman et al. (2010).

The mean LFP and MUA response magnitudes for each stimulus pulse from the 16 s, 5 Hz whisker stimulation are plotted in Fig. 6a. After the LFP response to the first stimulus pulse, the magnitude of subsequent responses to the train of stimulus pulses rapidly decreased and appeared to stabilise at approximately 70% of the first response magnitude, whereas the MUA responses to each stimulus pulse remained stable. To facilitate comparison of these neuronal response data obtained in awake animals with those obtained under similar experimental conditions in previous studies using anaesthetised animals (see Discussion below), in Fig. 6b we reproduce data previously reported in Boorman et al. (2010) with re-analysis to show the LFP and MUA responses to a 16 s, 5 Hz stimulation obtained under anaesthesia.

## Discussion

The main finding of this study is that monotonic stimulation of the contralateral whisker pad in awake rats leads to a complex spatiotemporal haemodynamic response function containing spatial and temporal signal components that are not present in data recorded from anaesthetised animals. This finding may have important implications for in-vivo studies which use anaesthetised animals for research into the neurovascular coupling and haemodynamic response mechanisms that underpin functional magnetic resonance imaging signals.

In previous studies with comparable spatial and temporal resolution, all of which have been conducted in anaesthetised animals, the haemodynamic response to a stimulus of this kind is characterised by a single response peak, without further oscillations (e.g. Ances et al., 2001; Berwick et al., 2005, 2008; Boorman et al., 2010; Devor et al., 2007; Dunn et al., 2005; Jones et al., 2002; Sheth et al., 2003); we confirm a response of this nature in the animals used in this study, once placed under anaesthesia after completion of imaging experiments in the un-anaesthetised state. The oscillations in the response time series recorded in awake animals under somatosensory stimulation conditions may be attributed to a number of factors. Firstly, we are able to rule out a likely contribution from additional response dynamics in the underlying neuronal activity, since we find that the magnitude profile of the neuronal responses to the stimulus train is very similar to that found in anaesthetised animals (see Fig. 6b and Ances et al., 2000; Berwick et al., 2008; Boorman et al., 2010; Martindale et al., 2005). Whilst the LFP and MUA measurements made for this purpose are not likely to be sensitive to all possible contributions from neuronal activity to the measured haemodynamic changes, LFP measurements in particular have been shown to be well correlated to haemodynamic changes and are generally considered an appropriate index of the cortical synaptic activity that drives neurometabolic and neurovascular responses (Logothetis et al., 2001). In this context therefore, the additional complexity of the awake animal haemodymamic responses appears likely to be driven by neurovascular or cerebrovascular factors, rather than by neuronal activity directly.

It is also important to rule out possible contributions from movement of awake animals to the responses observed, especially movement associated with stimulus delivery. We did not observe any stimulus-locked movement in our subjects whilst conducting the experiments, and additional analyses of the data (using cross-correlation methods on spatially interpolated data) reveal there was no image displacement during data acquisition (data not shown). Small movement may also lead to artefactual signal changes in the absence of detectable image displacement (for example small movement of the brain within the skull, or movement in the ‘z’ direction above and below the image plain). We have previously observed such movements in similar experiments using air-puff stimuli in awake animals. These are characterised by rapid-onset/offset stimulus locked signal changes, occurring within 500 ms of stimulus onset (Martin et al., 2002). Such signal changes are not evident in the response time series we report here with haemodynamic signal changes not becoming evident until ~ 750 ms. (Fig. 4a). This also suggests that the movements observed in our previous study were attributable to the acoustic component of the air-puff stimulus, a factor not present in the current study.

To explain the main findings of this work, we therefore suggest that the more rapid haemodynamic impulse response function found in the un-anaesthetised animal (Martin et al., 2006b) exposes response dynamics that are ‘temporally blurred’ by the longer time constant of haemodynamic responses in anaesthetised animals. The periodicity of the response oscillations (~ 2.5–3 s) is very similar to the time to peak of the haemodynamic response and the haemodynamic impulse response function for the somatosensory cortex of the un-anaesthetised rat (Martin et al., 2006b). It is uncertain whether the oscillatory response dynamics reported here are also a feature of responses in humans: the typical sampling rates of human BOLD fMRI studies are too low (~ 3 s per acquisition) to enable detection of these features. If present, these features could alter our understanding of the (un-anaesthetised) spatiotemporal haemodynamic response function and have substantial implications for the accuracy of biophysical models of neuronal-haemodynamic-fMRI processes and relationships (e.g. Zheng et al., 2010).

The observation of a continued increase in response magnitude as stimulation continues is also in contrast to most previous studies which have been conducted under anaesthesia (Ances et al., 2001; Boorman et al., 2010; Dunn et al., 2005; Martindale et al., 2005) as well as to the presently recorded data from animals anaesthetised after imaging sessions in the awake state. One exception is Jones et al. (2002), who investigated the responses to extended electrical stimuli of varying amplitude and demonstrated (in anaesthetised animals) that the initial response peak was succeeded by a decrease towards baseline (under continued stimulation) for lower amplitude stimuli and an increase under higher amplitude stimuli similar to that observed here for awake animals. We suggest that whereas lower amplitude neuronal activations are met with an overly large initial hyperaemic response which subsequently declines in magnitude as it becomes better matched to metabolic demand, larger activations are met with an inadequate initial hyperemic response which subsequently needs to ‘catch up’ with metabolic demand. The relatively more robust cortical responses to mild sensory stimuli in un-anaesthetised animals (e.g. Martin et al., 2002; Peeters et al., 2001) may thus lead to haemodynamic responses that continue to increase in magnitude despite steady state stimulus amplitudes and neuronal activity. We also speculate that this ‘sustained increase’ component of the response, together with the temporal changes observed as the response returns to baseline is characteristic of ‘delayed compliance’ as described by (Mandeville et al., 1999), the prominence of which in these data may be related to the effects of anaesthesia upon the baseline viscoelastic properties of vascular smooth muscle (Behzadi and Liu, 2005; Cohen et al., 2002). More generally, it is possible that the effects of anaesthetic state on vascular and haemodynamic baseline parameters (e.g. Smith and Wollman, 1972; Strebel et al., 1995) as well as upon neuronal and neurometabolic baselines (e.g. Hyder et al., 2002) may also contribute to the differences in response dynamics reported between anaesthetic conditions.

Finally, an important consideration in this work is that of restraint-stress and we are mindful of the possibility of substituting the experimental confounds of anaesthetic agents with the (neuro)physiological effects of stress. As has been reported in a previous work (Martin et al., 2006b) using optical imaging and electrophysiological techniques with the same animal training and restraint regimen as used in the present work, indications of stress were minimal and animals appeared to habituate well to the apparatus. Vocalisations and resistance to handling rapidly abated after initial training sessions and there were no indications of stress-related chromodacryorrhoea secretion or abnormal behaviour associated with acute stress or that might precede development of learned helplessness. In work conducted in other laboratories using restrained rats for fMRI, physiological and corticosterone measurements indicate significant reductions in stress after 5–8 days of training (King et al., 2005). Together with the lack of movement artefacts in our data (see above), these points suggest that stress was effectively minimised by our training and restraint procedures. We do however recognise that non-invasive monitoring (e.g. non-invasive pulse oximetry and blood pressure measurements) would be a useful addition to future work using this method.

## Conclusions

In conclusion, the present data reveal a number of features in the haemodynamic responses to sensory stimulation of un-anaesthetised rats that are not present in the previous work which has used anaesthetised animals. Additional studies in which neuronal and haemodynamic responses are measured concurrently in un-anaesthetised animals over a broader range of stimulus parameters are now required to determine spatiotemporal parameters of the neurovascular coupling relationship under these experimental conditions. These results suggest that anaesthesia has a significant impact on the spatiotemporal haemodynamic response function and further research is required to determine the significance of this for the applicability of findings from studies in anaesthetised animals to neuroimaging studies in awake humans and animals.
